# Public health consequences of armed conflict in Sudan in the face of global donor fatigue

**DOI:** 10.1002/puh2.156

**Published:** 2024-01-30

**Authors:** Muhammad Kabir Musa, Gibert Eshun, Mohamed Abdulkareem Adam Modber, Usman Abubakar Haruna, Abdullateef Abdulsalam, Asma'u Shuaibu Zailani, Farida Zakariya, Nuruddeen Abubakar Adamu, Mohamed Babiker Musa, Godness Kye Biney

**Affiliations:** ^1^ Department of Medicine Nazarbayev University School of Medicine Astana Kazakhstan; ^2^ Seventh‐Day Adventist Hospital Agona‐Asamang Ghana; ^3^ Faculty of Nursing Sciences University of Khartoum Khartoum Sudan; ^4^ Department of Biomedical Sciences Nazarbayev University School of Medicine Astana Kazakhstan; ^5^ Faculty of Pharmaceutical Sciences Ahmadu Bello University Zaria Nigeria; ^6^ Faculty of Pharmaceutical Sciences University of Abuja Abuja Nigeria; ^7^ Division of Experimental Medicine McGill University Montreal Canada; ^8^ Faculty of Pharmacy Omdurman Islamic University Khartoum Sudan; ^9^ Department of Biostatistics and Epidemiology School of Public Health and Health Sciences, University of Massachusetts Amherst Amherst Massachusetts USA

**Keywords:** armed conflict, disease outbreaks, donor fatigue, healthcare infrastructure, public health, Sudan

## Abstract

Sudan, a country located in northeastern Africa, is grappling with a profound health crisis resulting from the recent war that erupted on April 15, 2023. This conflict has inflicted significant damage on the country's health system, particularly through the destruction of healthcare infrastructure. Approximately 61% of health facilities have been destroyed, leaving only 16% operating optimally in Khartoum. Hospitals and clinics, vital for public health, have become targets of violence, exacerbating the challenges faced by the nation. The World Health Organization has noted the closure of roughly 16 hospitals since the start of the conflicts due to staff safety concerns as well as a shortage of hospital supplies, consumables, and medication. There has also been a gradual waning of donor support and resources allocated to address protracted crises and emergencies worldwide Sudan receives very little funding from donor organizations to maintain its healthcare system, which worsens the nation's general public health architecture. This makes the country vulnerable to serious challenges like disease outbreaks, starvation, infectious diseases, deteriorating health infrastructure, and mental health issues. To successfully reduce the severity of negative impacts on public health, the crisis must be ceased and facilities reopened. An emergency disease surveillance system for infectious diseases should be established, women and child health should be prioritized, and mental health awareness programs and services should be implemented. The global community must support the efforts to mitigate the devastating effects of this crisis. This article aims to highlight the critical impact of the recent war on Sudan's healthcare, advocating for urgent measures, including facility reopening, disease surveillance, and global support to mitigate devastating consequences.

## INTRODUCTION

Fragile, conflict‐affected, and vulnerable (FCV) settings are home to an estimated 1.8 billion people, as reported by the World Health Organization (WHO) [1]. These settings are often characterized by political instability, violence, poverty, and limited access to essential services such as healthcare. In recent times, several countries, such as Syria, Yemen, the Tigray Region of Ethiopia, and Ukraine, have been in the throes of armed conflicts, exacerbating the challenges the populations residing in these regions face. Recently, Sudan has been experiencing its share of armed conflicts, which is believed to be a result of a power tussle between the military and paramilitary groups. The power‐sharing agreement, inked in July 2019 between the army and a paramilitary group known as the Rapid Support Forces (RSF), brought a glimmer of hope for enduring peace and stability in the country. However, this hope proved to be short‐lived as the capital city of Sudan, Khartoum, became the epicenter of a renewed conflict between these opposing forces, which erupted on April 15, 2023, resulting in at least 559 fatalities [2] and more than 4000 injuries [3]. Armed conflict has been identified as one of the six hazards that cause 80% of health emergencies in the country, along with measles, cholera, dengue fever, malaria, and floods [4]. Sudan recently experienced a flood that resulted in massive displacement of people and the destruction of over a thousand latrines and homes. Access to clean water and hygiene was compromised, resulting in high incidences of several diseases, including the hepatitis E virus, dengue fever, and Rift Valley fever in Sudan and neighboring South Sudan [4, 5].

As the crisis worsens, the WHO Eastern Mediterranean Office reported an attack on the medical staff, health facilities, and ambulances, exacerbating the public health crisis of the already constrained healthcare system of the nation [6]. The anticipated effects of this crisis on the healthcare system include disease outbreaks, food scarcity, declining health infrastructure, and mental health challenges, among other issues, all of which are exacting a toll on the people of Sudan [7]. Sudan is among the African nations with few external donations, possibly due to political instability and ongoing conflicts that have raised security concerns and deterred donor agencies from providing aid. The political unrest, coupled with a weak economy and natural calamities, has further confounded the situation, resulting in a potential long‐term public health crisis. This article aims to highlight the critical impact of the recent war on Sudan's healthcare, advocating for urgent measures, including facility reopening, disease surveillance, and global support to mitigate devastating consequences.

## PUBLIC HEALTH IMPACT OF SUDAN'S CRISIS

The intricate relationship between health and peace is widely acknowledged within academic and policy circles, as peace is not only regarded as an essential precondition but also the foremost determinant for achieving optimal health outcomes [1]. It is stated, “There cannot be health without peace, and there cannot be peace without health” [6]. Conflicts and wars that disrupt peace inevitably inflict detrimental effects on the well‐being of individuals, communities, and the global population at large. Given the ongoing situation in Sudan, it is imperative to recognize the profound implications it carries for both the health of its population and global public health, warranting our utmost attention and concern.

The current war is disintegrating and collapsing the health system of the country, which might take decades to rebuild. The health system in the country was already severely underfunded, with inadequate human resources, infrastructures, and essential medicines; a doctor‐to‐population ratio was 4:10,000 [8], and 70% of health facilities were lacking essential lifesaving medical products [4]. This fragile healthcare system has been weakened more, as seen in the early weeks of the armed conflict, when there has been destruction of 61% of all health facilities with only 16% operating at optimum in Khartoum [9]. These destructions of facilities, together with interruption and shortages in medical supplies and sudden exodus of healthcare workers, will lead to disruption and cessation in service provision, affecting access to healthcare [10]. This comes at the detriment of both patients with acute and chronic illnesses. Chronic patients with diseases like hypertension will forfeit treatments, leading to disease‐related complications and deaths. Furthermore, the cessation of essential child health services such as immunization and maternal health services such as skilled birth attendants (SBA) comes with dreadful consequences. Sadly, an estimated 24,000 women in Sudan due to give birth in coming weeks will be unable to access SBA [9], increasing the maternal deaths, stillbirths, and infant mortality in the country. This is an impediment to the achievement of SDG 3, and it reaffirms why over half of the unmet needs for key target areas such as maternal and child mortality of SDG 3 occur in FCV countries [1].

The ongoing crisis in Sudan has the propensity to cause epidemics, particularly those that are infectious in nature and could devastate the country, worsen healthcare services, and pose a global health threat in the third‐largest African nation by population. It has been found that 70% of disease outbreaks that WHO responds to take place in FCV settings [1]. Sudan is no different, considering the events unfolding in the country. Currently, the National Public Health Laboratory in Khartoum is being used as a base by the fighters [9]. This lab houses dangerous pathogens like measles, TB, cholera, polio, and SARS‐CoV‐2. Exposure to these pathogens risks outbreaks in Sudan and beyond, as migration could spread diseases where immunization is lacking. This could have massive consequences by starting epidemics in a region ill‐prepared to handle spreading infectious diseases [6]. Outbreaks of diseases like polio may derail all achievements made in their control and elimination. Furthermore, access to these deadly pathogens by the desperate fighters brings to mind the possibility of bioweapon and bioterrorism. Furthermore, the massive internal displacement and cross‐border movement make room for water, sanitation, and hygiene (WASH)‐related diseases to exploit and flourish. Displaced persons are at increased susceptibility for WASH‐related diseases due to risk factors, such as overcrowding, poor sanitary conditions, and a lack of clean water. It has been found that people in conflict‐affected areas are three times as likely to practice open defecation, four times as likely to lack basic sanitation services, and eight times as likely to lack basic drinking water [11].

Mental health problems resulting from population displacement and disconnection from loved ones, such as post‐traumatic stress disorder, mood disorders, depression, suicidal behavior, and behavioral problems, arise from the traumatic experiences during conflicts that can last for lifetime. One out of five people living in conflict areas face mental illness [12]. Moreover, women and children living in and near conflict‐affected areas become easy prey for harassment, assault, sexual violence, early marriage, exploitation, and trafficking [12]. Food insecurity arises in and near areas of conflicts sending millions of people into starvation. This is seen in Ethiopia and Yemen where, in 2020 alone, food costs soared up to 50% [12]. This is particularly devastating for Sudan, considering that prior to this recent event, 11.7 million people [a quarter of the population] were facing acute hunger, 3.1 million people were at the emergency level of food insecurity, and over 3 million under 5 children were suffering from acute malnutrition with 650,000 of them being severe acute malnutrition [13]. The effect of food shortage will not only be limited to Sudan but also to surrounding countries like Chad, which relies heavily on imported food products landing at the port in Sudan. It will inevitably lead to increased malnutrition in the region, which, coupled with other factors, will rob people, particularly children of quality health, which in turn affects their health and productivity in adulthood. Furthermore, armed conflict is a major contributing factor to poverty, which affects health and healthcare access. It is not surprising that estimates show that by 2030, at least half of the world's poor people will be living in FCV countries [1].

**FIGURE 1:**
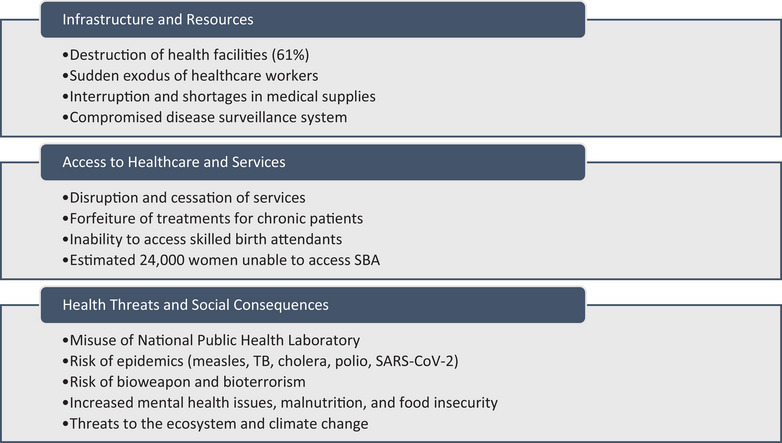
Impact of armed conflict on Sudan's healthcare system.

Beyond the direct health impact on humans are the terrible consequences on the environment and ecosystem, which take decades to reverse. Air, soil, and water pollution are common environmental effects of armed conflicts arising from the use of ammunition (guns and bombs), heavy vehicles, the destruction of forests, and many other armed conflict‐related activities as summarized in Figure 1. These consequences have a long impact on the ecosystem and contribute to threats like climate change. Hence, safeguarding the right to health for individuals is essential, as it also entails preserving the integrity of our environment and ensuring the well‐being of our planet for future generations [14].

## GLOBAL DONOR FATIGUE

Navigating the challenges of Sudan's crisis is further compounded by the phenomenon of global donor fatigue, which carries significant implications for the country and its humanitarian situation. Over time, there has been a gradual waning of donor support and resources allocated to address protracted crises and emergencies worldwide [15]. This trend has been attributed to a variety of factors, including competing humanitarian crises, economic constraints, and a sense of exhaustion among donors.

The implications of global donor fatigue for Sudan's crisis are far‐reaching. As the country grapples with pressing humanitarian needs, such as the provision of healthcare, food security, clean water, and shelter, the reduced availability of funds and resources poses a serious challenge to the efforts aimed at alleviating the suffering of its population [16]. The consequences are particularly dire for vulnerable groups, including women, children, and internally displaced persons, who heavily rely on external assistance for their survival.

Prior to the conflict, an estimated 15.8 million people in Sudan (32% of the population) needed humanitarian assistance, with 11 million of them requiring emergency aid for life‐threatening conditions related to health and also to meet the minimum standards of living essential for good health [4]. Due to the conflict, there is an escalation of this assistance, which has now been catastrophic and is reaching a breaking point. With the ongoing fighting, agencies are finding it difficult to deliver aid to meet these humanitarian needs required for good health and the survival of the population. Disturbingly, Sudan is home to over 1 million refugees and serves as a source, transit point, and final destination for mixed migrations of migrants, refugees, and asylum seekers from all over the African subregion traveling to Europe and other countries [6].

## RECOMMENDATIONS FOR RESTORING HEALTH

The immediate and long‐term impact of conflicts and wars on population and global health is immense and therefore cannot be overlooked. As such every effort must be made by the opposing parties and international bodies to bring peace to Sudan. In the short term, both parties must respect the ceasefire periods to enable humanitarian agencies to deliver aid to the suffering victims of the country. As many people are being displaced, refugee camps should ensure that factors that make WASH‐related diseases thrive are reduced to the minimum. The government should prioritize and reorganize regional health centers across all affected areas with well‐equipped facilities, staff, and medical supplies, while also enacting comprehensive bye‐laws to protect healthcare workers.

A well‐structured disease surveillance system is necessary to prepare for infectious disease outbreaks. Telemedicine offers a range of solutions that can address challenges, such as disease outbreaks, food scarcity, deteriorating health infrastructure, and mental health issues. By incorporating telemedicine platforms, Sudan can enable virtual consultations, remote patient monitoring, and e‐pharmacy services, ensuring access to medical care and medications even in difficult circumstances. Additionally, telemedicine can support health education, mental health services, and knowledge exchange between local healthcare practitioners and global experts. The integration of telemedicine data can aid in tracking disease trends and informing evidence‐based decision‐making.

The government should also focus on addressing malnutrition in children under five by providing dietary support, free healthcare for pregnant women and young children, and establishing special mental health facilities with trained personnel to address mental health issues caused by the ongoing crisis.

## CONCLUSION

Conflict and crisis, in any shape or form, are calamities that result from human actions. The sanctity of human life should be upheld by those in power. The ongoing crisis in Sudan is a challenging period for its people, and prompt action is necessary to prevent further deterioration of the nation's public health status, both in the present and in the long term. The global community must act expeditiously to mitigate the devastating effects of this crisis.

## AUTHOR CONTRIBUTIONS

*Muhammad Kabir Musa: Conceptualization; Writing – review & editing; Writing – original draft. Gibert Eshun: Conceptualization; Writing – original draft; Writing – review & editing. Mohamed Abdulkareem Adam Modber: Writing – review & editing; Validation. Usman Abubakar Haruna:Conceptualization; Writing – original draft; Writing – review & editing; Supervision; Validation. Abdullateef Abdulsalam:Conceptualization; Writing – original draft; Writing – review & editing. Asma'u Shuaibu Zailani: Writing – review & editing; Writing – original draft. Farida Zakariya: Writing – review & editing; Writing – original draft. Nuruddeen Abubakar Adamu: Writing – original draft; Writing – review & editing. Mohamed Babiker Musa: Validation; Writing – review & editing. Godness Kye Biney: Writing – review & editing; Writing – original draft*.

## CONFLICT OF INTEREST STATEMENT

Usman Abubakar Haruna is a member of the Youth Editorial Board of Public Health Challenges and was not involved in any decision related to this manuscript.

## FUNDING INFORMATION

No financing is disclosed.
